# Structural and electronic effects of adatoms on metallic atomic chains in Si(111)5 × 2-Au

**DOI:** 10.1038/s41598-018-33703-5

**Published:** 2018-10-19

**Authors:** Eui Hwan Do, Se Gab Kwon, Myung Ho Kang, Han Woong Yeom

**Affiliations:** 1 Center for Artificial Low Dimensional Electronic Systems, Institute for Basic Science (IBS), Pohang, 37673 Republic of Korea; 2 Department of Physics, Pohang University of Science and Technology (POSTECH), Pohang, 37673 Republic of Korea

## Abstract

We investigate the effects of native Si adatoms on structural and electronic properties of the Si(111)5 × 2-Au surface, a representative one-dimensional metal-chain system, by means of scanning tunneling microscopy (STM) and density functional theory (DFT) calculations. High-resolution STM images of relatively long adatom-free chain segments evidence directly the inherent ×2 reconstruction, which is the essential part of a recently proposed structural model based on a renewed Au coverage of 0.7 monolayer. On the other hand, STM images for chain segments of different lengths reveal that the structural distortion induced by Si adatoms is confined in neighboring unit cells, in good agreement with DFT calculations based on that model. Si adatoms greatly affect the metallic bands of Au chains, one of which becomes fully occupied and represents a tightly confined electronic state to the distortion around Si adatoms, potentially forming short insulating segments within metallic chains. This finding provides an atomic-scale understanding of the observed gradual metal-insulator transition and atomic-scale phase separation induced by Si adatoms.

## Introduction

One-dimensional (1D) metal atomic chain systems on semiconductor surfaces have attracted substantial attention because of their intriguing electronic properties and potential applications in atomic scale devices. Various important fundamental issues have been discussed including non-Fermi liquid behavior^1^, charge density waves^2^, giant Rashba splitting^3^, Si magnetism^4^, nontrivial topology^5^, and topological excitations^6^. Atomic chain structures on flat and vicinal Si (or Ge) surfaces induced by In or Au adsorbates are the most actively discussed, and the Si(111)5 × 2-Au surface has been the model system for the latter cases^7–11^. This surface has been extensively investigated by both experimental^7,11–22^ and density functional theory (DFT) studies^23–26^ since 1980’s. These works revealed interesting properties related to its 1D metallic band structure. Intriguingly, this particular surface phase cannot be fabricated without a substantial density of Si adatoms. The adatom density can be even controlled globally^11^ or in an atom-by-atom fashion^17^, which, in turn, makes the physics landscape richer with the adatom-related metal-insulator transition^11,27,28^, 1D electron confinement^22^, and topological defects^19^. However, the microscopic understanding of the effects of Si adatoms onto the structural and electronic properties has not been fully established.

**Figure 1:**
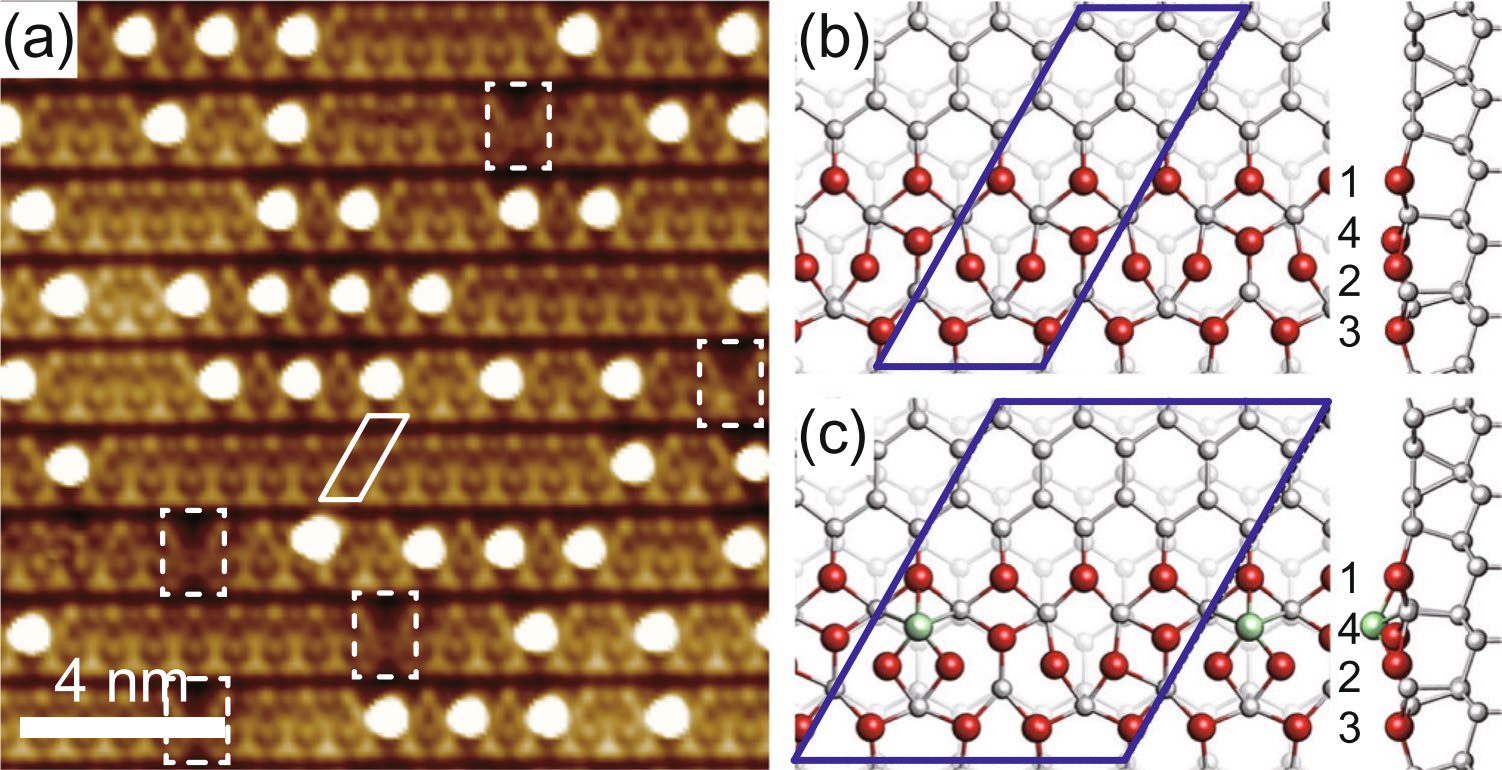
(**a**) Topographic STM image (15 × 15 nm^2^) of Si(111)5 × 2-Au at 78.2 K. Bright protrusions are Si adatoms formed inevitably during surface preparation. Bias voltage and tunneling current are −1.0 V and 300 pA. A 5 × 2 unit cell (solid lines) and atomic-scale dislocations^19^ (dashed lines) are indicated. KK^26^ models for the (**b**) adatom-free and (**c**) 0.05 monolayer Si adatom (4*a*_0_ chain) surfaces. Au (Si) atoms are represented by large (small) balls. Different Au atomic rows are indicated as numbers.

Moreover, the atomic structure of the Si(111)5 × 2-Au surface itself had been elusive with various structural models of different Au concentration proposed^15,16,23–26,29,30^. A conclusive structural model was only recently proposed by Kwon and Kang^26^ (hereafter, KK model), which was then solidly supported by quite a few updated experiments^11,12,26,28,31–33^. Within the KK model, the characteristic ×2 reconstruction of the atomic chains is realized by the intrinsic ×2 arrangement of Au atoms (see Fig. 1) in clear contrast to the previous model, which resorts to the global doping effect of Si adatoms^25^. However, some extraordinary features such as metal-insulator transition observed especially at high adatom density^11,26,28^ could not be fully understood. A simple explanation of this electronic transition was the energetic benefit by the band gap opening due to a ×4 adatom superlattice [Fig. 1(c)]^25,26,28^. Such approach, however, emphasizes only the global effect of a periodic adatom arrangement while neglects largely the local actions of individual adatoms such as the strong potential barrier observed experimentally^22^. Therefore, further investigation is needed on the local effects of a Si adatom to nearby atomic and electronic structures. These local modulations are expected to act more dominantly as the adatom coverage increases, in consequence, would become important for the intriguing change of the band structure and the electronic phase transition at high adatom density^11,26,28^.

In this work, we investigate the effects of Si adatoms on the Si(111)5 × 2-Au surface by examining local atomic and electronic structures around the Si adatoms with the use of scanning tunneling microscopy (STM) measurements and DFT calculations. The intrinsic ×2 reconstruction of the pristine Au chain^26^ is evidenced directly by our STM images on long adatom-free chain segments, which is essential in the KK model. Si adatoms are found to little affect the substrate Au-chain structure; the adatom induced structural distortion occurs only in neighboring unit cells. Si adatoms, however, greatly affect the metallic bands of the Au chains; one of the metallic bands becomes fully occupied just below the Fermi energy and represents a tightly confined electronic state around the Si adatom. This possibly accounts for the intriguing metal-insulator transition and atomic-scale phase separation observed with the increase of Si adatoms^11,28^.

## Results and Discussion

Figure 1(a) shows a high resolution topographic STM image of the Si(111)5 × 2-Au surface. The 5 × 2 chain structure is evident along the horizontal direction. The atomic wires are decorated and segmented by Si adatoms which appear as strongly bright protrusions. Most of wire segments have their length (distance between neighboring adatoms) of even multiples^34^ of *a*_0_ [*a*_0_ = 0.384 nm, the Si(111) lattice constant]^19,26^. As discussed in detail below, the adatom-free parts of the segments have a 2*a*_0_ period structure everywhere. The only exception is the rarely observed segments with a length of odd multiples of *a*_0_, each of which contains one characteristic 3*a*_0_ dislocation^19^ [dashed squares in Fig. 1(a)]. The length distribution among even-length segments follows the Poisson behavior^19^ resulting in a predominant number of short segments^19,34^. Since it has been practically impossible to access the sufficiently low adatom coverage condition in measurements, which would contain adatom-free segments predominantly^11^, the previous structural models including the KK model were primarily discussed based on their consistency with the band structure data^11^ of the highest adatom coverage corresponding to a ×4 adatom superlattice^25,26^. It is thus essential to systematically compare the experimental and theoretical STM images of segments with different lengths in order to clearly understand the role of Si adatoms in atomic and electronic structures.

Figure 1(b,c) illustrate the schematics of the KK model for the clean surface of Si(111)5 × 2-Au and that with a ×4 superlattice (0.05 monolayer of Si adatoms), respectively. Au wire bundles are separated by Si honeycomb chains to form 1D electronic bands^35^. The KK model has additional Au atoms located between rows 1 and 2 in an interval of 2*a*_0_ along wires (constructing row 4). This results in a ×2-period distortion in neighboring Au and Si rows between rows 2 and 3. The Si adatom prefers to adsorb on a hollow site in row 4 [Fig. 1(c)] without a significant change in the underlying (5 × 2) atomic arrangement^26^.

**Figure 2:**
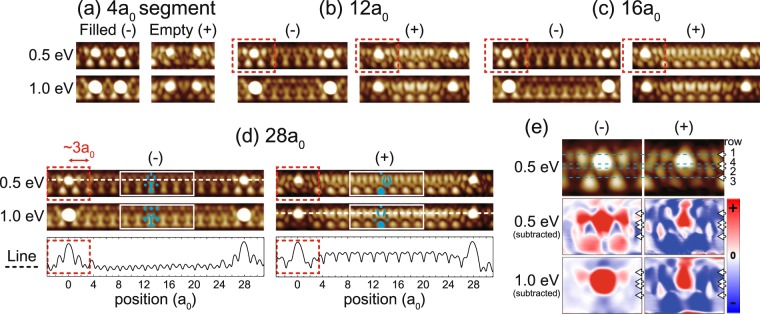
Bias-dependent STM images of (**a**) 4*a*_0_, (**b**) 12*a*_0_, (**c**) 16*a*_0_, and (**d**) 28*a*_0_ wire segments. Filled and empty state images are taken at ± 0.5 and 1.0 V biases. Characteristic features are marked by blue solid lines and symbols. The distorted structure next to the adatoms is indicated by square (dashed). (**e**) Bias-dependent images around a Si atom and those after being subtracted by the corresponding images of the adatom-free part in order to show the adatom contribution more clearly. The adatom-free topography is taken from the central part of a 28*a*_0_ segment. Red (blue) color indicates enhancement (suppression) of the contrast by the adatom.

The atomic structure is examined with the bias-dependent STM images of wire segments with lengths of 4*a*_0_, 12*a*_0_, 16*a*_0_, and 28*a*_0_ [Fig. 2(a–d)]. One can clearly notice the ×2 reconstruction appearing as well-known Y- and V-shaped features in filled and empty states, respectively^19,25,26^ [Fig. 2(a–d)], even for a very long segment of 28*a*_0_. This corresponds to a fairly low adatom coverage of ~0.007 monolayer. While there is a structural distortion due to a Si adatom, its extent is limited only to 3*a*_0_ (1.5 unit cells) from the center of the adatom as indicated in Fig. 2(d). This unambiguously demonstrates the inherent nature of the ×2 period structure.

**Figure 3:**
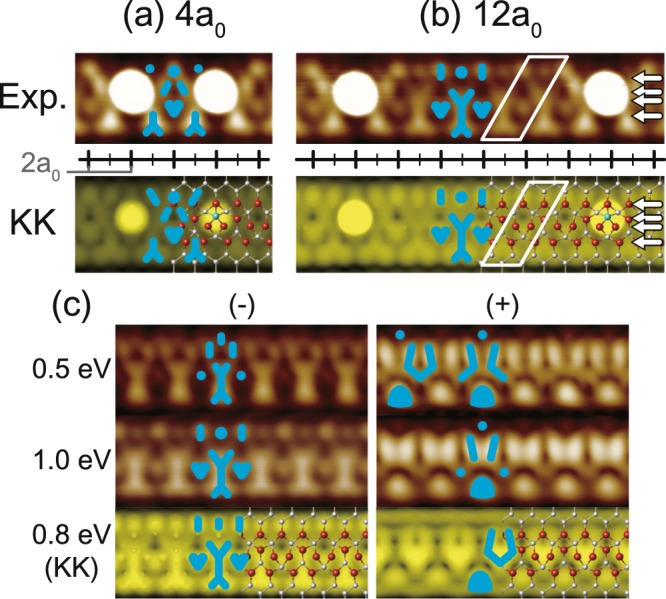
Topographic STM images of (**a**) 4*a*_0_ and (**b**) 12*a*_0_ wire segments, taken at bias voltage −1 V (filled states). The corresponding simulated STM images based on the KK model are shown for direct comparison. Solid boxes depict the smallest repeated structures. (**c**) The simulated STM images (the KK model at an intermediate bias) of the adatom-free surface are directly compared with experimental ones taken at the central part of the longest wire segment (indicated in Fig. 2(d)). ×2 periodic Λ- and Y-shaped features are marked by blue solid line and symbols.

The atomic and electronic structures of wire segments with different lengths are also investigated by DFT calculations. We show the filled state STM images and corresponding DFT simulations of 4*a*_0_ (the shortest) and 12*a*_0_ segments representatively in Fig. 3(a,b). For the shortest segment of 4*a*_0_, the ×2 bright distortion appears commonly for the experiment and the simulation as a Λ-shaped protrusion between two neighboring adatoms. This bright distortion in the STM images of a 12*a*_0_ segment, however, extends only to 3*a*_0_ from the adatom center, and the other parts of the chain exhibit Y-shaped features very similar to the adatom-free structure [Fig. 3(c)]. This result clearly indicates that the Si-adatom-induced effect, either electronic or structural, is well confined spatially. Therefore, this result directly denies the possibility of periodic transitions due to the global chemical potential change through the adatom-induced electron doping, which are prevalently observed in similar Au-induced chain systems^36^. The present result is fully consistent with the recent scanning tunneling spectroscopy (STS) study indicating the absence of any substantial chemical potential change induced by adatoms on the adatom-free parts of chains^22^.

Then, we scrutinize the ×2 reconstruction of the adatom-free part of the surface. In order to do this, we pick up the longest segment observed in the experiment with a length of 28*a*_0_ [Fig. 3(c)]. The 28*a*_0_ wire segment is sufficiently long to clarify the structure of the pristine wire as clearly confirmed by the detailed line profiles of the STM topography [Fig. 2(d)]. The unperturbed ×2 periodic Y- and V-shaped features are well matched with the DFT simulations of the KK model in both filled and empty states. In addition to the agreement of the KK model prediction with all other available spectroscopic and global structural data, the present work firmly corroborates the consistency between the experiment and model. Especially, our conclusion is free from any ambiguity provided by Si adatoms, which was not the case with most of the previous experimental studies.

**Figure 4:**
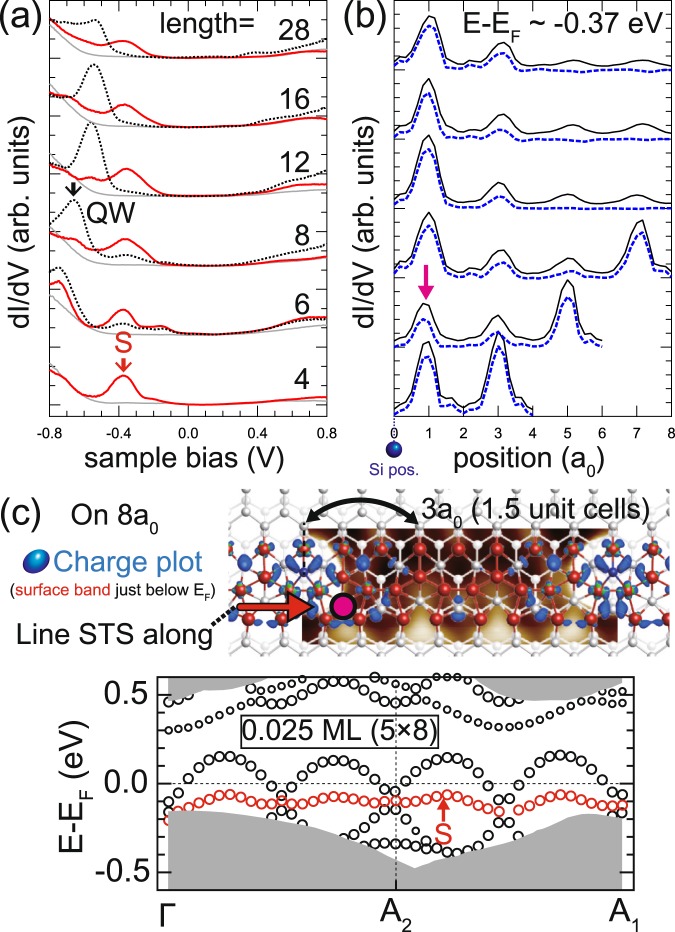
(**a**) *dI*/*dV* curves of 4*a*_0_, 6*a*_0_, 8*a*_0_, 12*a*_0_, 16*a*_0_, and 28*a*_0_ wire segments. Red curves are recorded at positions 1*a*_0_ away from Si adatoms [magenta markers in (**b**) and (**c**)] and vertical offsets are given for comparison. Gray and dotted line curves display reference data sampled on Si adatom and center of chain positions^22^, respectively. (**b**) *dI*/*dV* spatial profiles measured at a bias of −0.37 V from the adatom center up to the 8*a*_0_ apart positions of the selected wire segments. Dashed profiles display the same profiles after subtracting the background ×2 modulation of the adatom-free part. (**c**) Charge plot and associated band structure of the surface with 0.025 monolayer adatoms (5 × 8)^26^. STM image of 8*a*_0_ segment is taken at bias voltage −1 V. The charge density (blue area) calculated from the surface state band below Fermi level (red circles) is overlaid. The calculated band dispersion of the 5 × 8 structure are also given, where the smaller circles indicate less weights in the topmost layer.

Locally distorted structures around adatoms are observed for different biases and segment lengths [Fig. 2(a–d)]. Topographic images of the distortion [Fig. 2(e)] exhibit strong bias dependence; the distortion develops into bright protrusions throughout the rows 1–4 at −0.5 eV. This apparently indicates significant local density of states (LDOS) variation around the Fermi level or the existence of a well localized electronic state. In Fig. 4(a), *dI*/*dV* curves of the distorted structures are shown^22^ for wire segments with lengths of 4*a*_0_, 6*a*_0_, 8*a*_0_, 12*a*_0_, 16*a*_0_, and 28*a*_0_. The detailed plots of the *dI*/*dV* spectra around the Fermi energy are shown in the Supplementary Information [Fig. S1]. One prominent spectral feature, the LDOS maximum labeled as *S*, is observed commonly at −0.37 V sample bias beneath Fermi level regardless of the segment length. In contrast to one dimensional quantum well (QW) states spreading over a segment^22^, the *S* state does not show any noticeable length-dependent energy shifts and decays rapidly from adatoms [Fig. 4(a,b)]. The lateral extent of the *S* state is about 3*a*_0_ from the adatom and well matched with that of the structural distortions in topographic images. Hence, the origin of the *S* state is well correlated to the distorted structures. One remarkable finding is that the *S* state is promoted to one of the most dominant states in the electronic structures of the shortest (4*a*_0_) and the second shortest (6*a*_0_) segments. This suggests that the band structure of the short segments would be largely determined by the electronic state of the distorted regions.

The strongly localized nature of the adatom-induced *S* state demonstrates the role of Si adatoms on the surface. In the calculation, Si adatoms are shown to form covalent bonds with the substrate surface atoms to make a fully filled band *S*. The chemical potential tuning of the adatoms, however, is rather limited around them as presented in the charge plot in Fig. 4(c). This indicates that the adatoms donate electrons to the neighboring structural distortions but not further into the host bands of unperturbed parts of the Au wires (black circles), which consistently explains the observed conductance difference between those two parts [Fig. S1]. The detailed discussion on this is provided in the Supplementary Information [Figs. S2 and S3]. That is, the *S* band is well decoupled from the host metallic band of the adatom-free part of the surface. Thus, the surface becomes a mixture of insulating short segments (or linear adatom clusters) with the *S* state dominating and longer metallic segments. This explains the very local phase separation into metallic and insulating segments observed previously^27^. At a high adatom coverage, the short segments dominate the surface, which would become overall insulating. This explains the gradual change of the band structure from metallic into insulating one in angle-resolved photoemission spectroscopy (ARPES) measurements upon increasing the adatom coverage^11,28^. That is, the apparent metal-insulator phase transition is due to the local phase separation and the gradual change of the phase population. The present experimental and theoretical studies explain the intriguing electronic properties of the surface in a unified way, the local electronic phase separation and the gradual evolution of the bands. Note that the adatom-induced state in the calculation [Fig. 4(c)] has a lower binding energy by about 0.2 eV than the present STS data and the previous ARPES data^11^. The discrepancy in the calculation possibly arises from the ambiguity due to the adatom-free segment which forms a quarter of the ×8 adatom superlattice [Fig. 4(c)]. The binding energy of the adatom-induced state, instead, is predicted more correctly by the calculation for the fully distorted segment (×4) which leads to an insulating band structure^26^. Nevertheless, the present analysis confirms solidly the adatom-induced occupation of one metallic band of the Au chain that produces a fully filled and strongly confined electronic state around the adatoms.

## Conclusion

In this combined STM and DFT study, we examine the structural and electronic effects of Si adatoms on the Si(111)5 × 2-Au surface. Firstly, the ×2 structure of adatom-free Au chains is confirmed directly by the STM measurements on long chain segments. We show that the details of STM images around Si adatoms and of adatom-free parts agree well with the DFT calculations based on the KK structural model. In addition, Si adatoms lead to the structural distortion only in neighboring unit cells and make one metallic band of the Au chain fully occupied to induce a tightly confined electronic state around them with negligible global effects. This well localized and fully filled electronic state accounts well for the intriguing electronic properties induced by adatoms such as the atomic scale electronic phase separation and the gradual metal-insulator transition.

## Methods

### Sample preparation

In order to prepare clean Si(111)7 × 7 surfaces, the Si(111) substrate was repeatedly flash-heated to 1520 K. 0.6–0.7 monolayer of Au was evaporated onto the clean surface held at 932 K to yield a well-ordered 5 × 2-Au surface^37,38^.

### STM measurement

All STM measurements were carried out with a commercial ultrahigh-vacuum cyrogenic STM (Unisoku, Japan) in the constant-current mode with tungsten tips at 78 K. The differential conductance, *dI*/*dV*, was measured using the lock-in detection with a modulation of 1 kHz.

### DFT calculations

DFT calculations were performed using the Vienna *ab initio* simulation package^39^ within the Perdew-Burke-Ernzerhof generalized gradient approximation^40^ and the projector augmented wave method^41,42^. The Si(111) surface is modeled by a periodic slab geometry with eight atomic layers and a vacuum spacing of about 11 Å. The calculated value of 2.372 Å is used as the bulk Si-Si bond length. Au atoms are adsorbed on the top layer of the slab, and the bottom layer is passivated by H atoms. We expand electronic wave functions in a plane wave basis with an energy cutoff of 250 eV. A (2 × 8 × 1) k-point mesh is used for the (5 × 2) Brillouin-zone integration. All atoms but those in bottom two Si layers are relaxed until the residual force components are within 0.02 eV/Å. Similar calculation schemes were successfully used in the previous work on the Si(111)-Au surface^26^.

## Electronic supplementary material

Supplementary information
