# Supplementary material for: Structural and electronic effects of adatoms on metallic atomic chains in Si(111)5 × 2-Au
Source: Sci Rep. 2018 Oct 19;8:15537. doi: 10.1038/s41598-018-33703-5 (PMC6195602; doi:10.1038/s41598-018-33703-5)
Supplement: Supplementary file 1 — Supplementary information [file 41598_2018_33703_MOESM1_ESM.pdf]

Supplementary information for "Structural and electronic effects of adatoms on metallic atomic chains in Si(111)5×2-Au"

Eui Hwan Do,[1,2,*] Se Gab Kwon,[2] Myung Ho Kang,[2] and Han Woong Yeom[1,2,†]

[1] *Center for Artificial Low Dimensional Electronic Systems, Institute for Basic Science (IBS), Pohang 37673, Republic of Korea*

[2] *Department of Physics, Pohang University of Science and Technology (POSTECH), Pohang 37673, Republic of Korea*

Conductance difference between distorted and adatom-free parts of the Au wire

Figure S1 shows detailed electronic structures around Fermi energy of a few selected dI/dV curves from Fig. 4(a) of the original paper. The selected wire segments with lengths of $4a_0$, $8a_0$, and $28a_0$ represent three distinctive surface structures: fully distorted one, partly distorted one with high adatom coverage, and nearly adatom-free one, respectively. On the fully distorted wire segment [Fig. S1(a)], dI/dV curves do not show strong local density of states (LDOS) around the Fermi energy except for the adatom-induced S state on the distorted part. Similar dI/dV curves are observed on the distorted structures of the $8a_0$ and $28a_0$ segments [Figs. S1(b) and (c)]. However, the center positions of those segments show apparently higher LDOS (dotted curves), which directly indicates higher conductance of the adatom-free parts of the Au wires than that of the distorted parts. This conductance difference between the distorted and adatom-free regions is well supported by the calculated charge distribution [Figs. S2 and S3].

Charge plots of various surface bands for 5×8 (0.025 ML Si-adatoms) and adatom-free surfaces

Charge analyses for the 5×8 (0.025 ML Si-adatoms) and adatom-free surfaces are shown in Figs. S2 and S3, respectively. On the 5×8 surface [Fig. S2], a fully filled band S is strongly localized to Si adatoms and their neighboring structural distortions with $3a_0$ lateral extent. In contrast, a metallic band M spreads over adatom-free parts (center) of the Au wire but vanishes in the distorted regions. This spatial distribution of the band M consistently explain the observed higher conductance of the adatom-free parts of the Au wire [Fig. S1].

On the clean 5×2 surface [Fig. S3], both bands B_1 and B_2 spread over pristine Au wire. The metallic band B_2 has similar charge spatial distribution to the band M in the adatom-free Au parts of 5×8 surface [Figs. S2(c) and S3(c)], which implies their identical origin of the pristine Au chain. The B_1, on the other hand, has qualitatively different charge distribution to the fully-filled band S although their binding energies are in close range [Figs. S2(b) and S3(b)]. This indicates that the origin of the band S is strongly related to the Si adatoms rather than the substrate Au chain. Therefore, the present charge analyses can transparently explain the role of Si adatoms on the Au chain; they donate electrons limitedly to very localized areas around them to induce the fully filled band S while do not interact noticeably with the metallic band M of the adatom-free parts of the chain.

* yggdrasil@postech.ac.kr

† yeom@postech.ac.kr

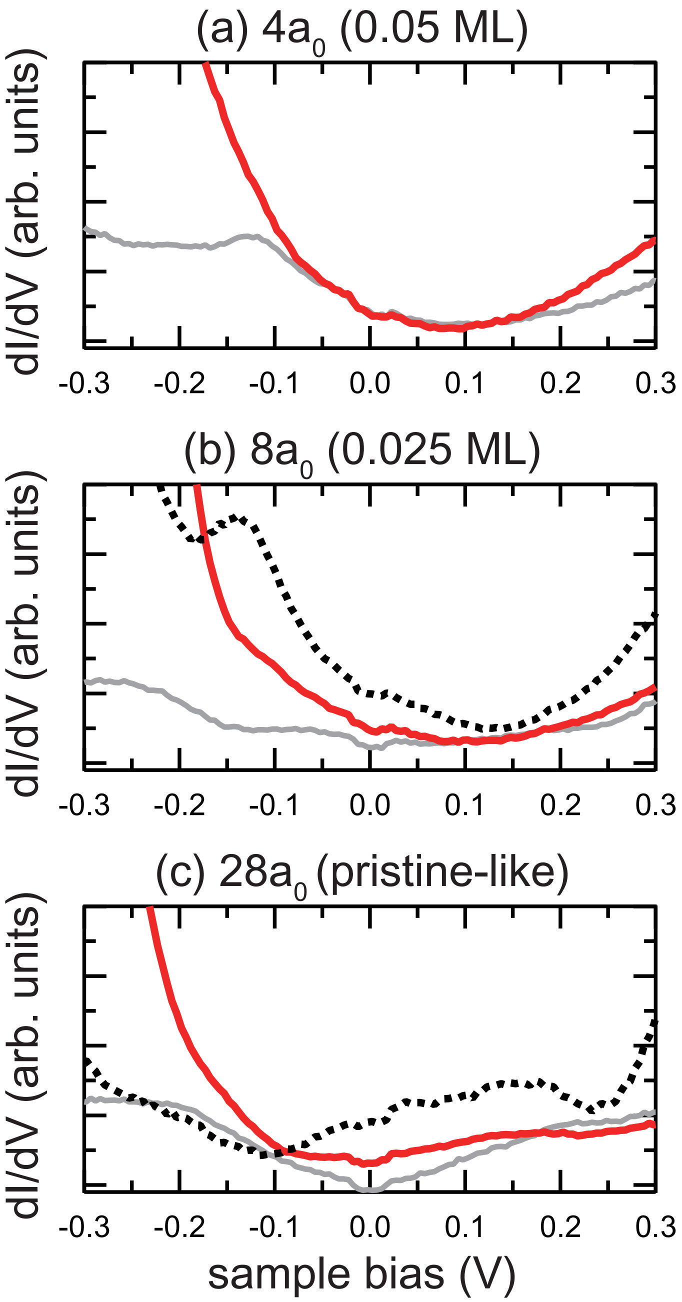

FIG. S1. Detailed features around the Fermi energy of dI/dV curves of (a) $4a_0$, (b) $8a_0$, and (c) $28a_0$ wire segments shown in Fig. 4(a) of original paper. Red curves are recorded at positions $1a_0$ away from Si adatoms and vertical offsets are given for comparison. Gray and dotted line curves display reference data sampled on top of Si adatom and center of chain positions, respectively.

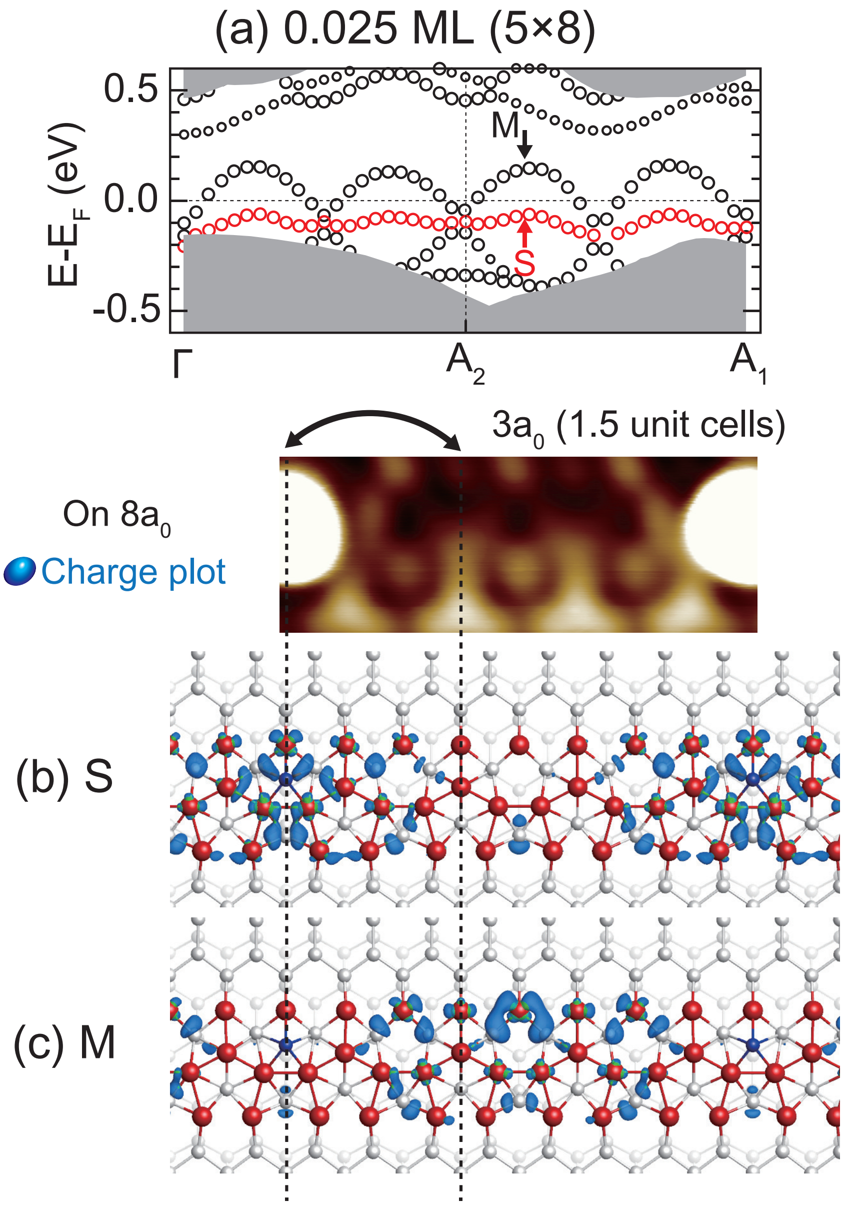

FIG. S2. (a) Band structure of the Si(111)5×8-Au surface with 0.025 monolayer adatoms. Calculated charge density (blue areas) of (b) S and (c) M bands are displayed with the topographic STM image of $8a_0$ segment (at -1.0 V bias) in same position. Smaller circles and shaded areas represent the surface band with less weights in the topmost layer and the bulk band, respectively.

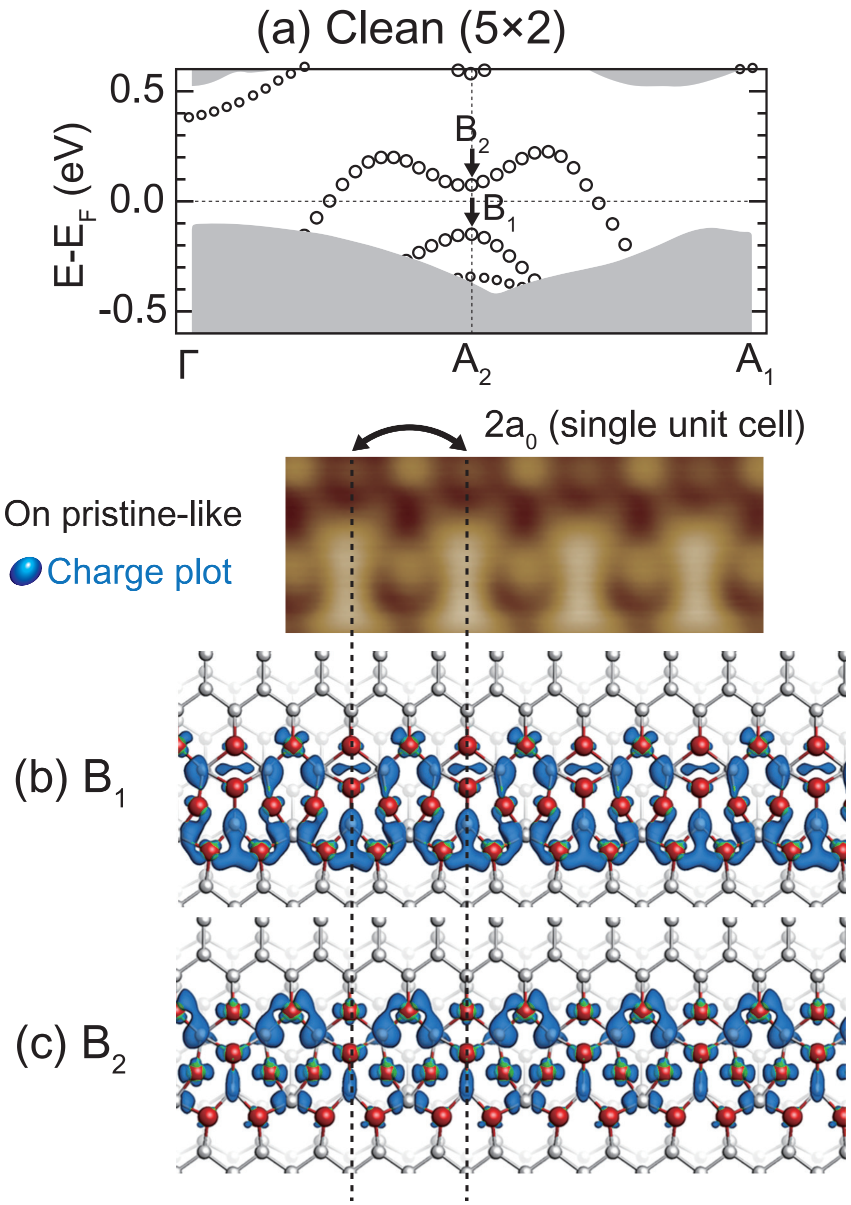

FIG. S3. (a) Band structure of the adatom-free Si(111)5×2-Au surface. Calculated charge density (blue areas) of (b) B_1 and (c) B_2 bands are displayed with the topographic STM image of pristine-like $28a_0$ segment (at -1.0 V bias) in similar position. Smaller circles and shaded areas represent the surface band with less weights in the topmost layer and the bulk band, respectively.
